# Clinical and Preclinical Evidence for M_1_ Muscarinic Acetylcholine Receptor Potentiation as a Therapeutic Approach for Rett Syndrome

**DOI:** 10.1007/s13311-022-01254-3

**Published:** 2022-06-07

**Authors:** Mackenzie Smith, Bright Arthur, Jakub Cikowski, Calista Holt, Sonia Gonzalez, Nicole M. Fisher, Sheryl Anne D. Vermudez, Craig W. Lindsley, Colleen M. Niswender, Rocco G. Gogliotti

**Affiliations:** 1 Department of Molecular Pharmacology and Neuroscience, Loyola University Chicago, Maywood, IL 60153 USA; 2 Edward Hines Jr. VA Hospital, Hines, IL 60141 USA; 3 Department of Pharmacology, Vanderbilt University, Nashville, TN 37232 USA; 4 Warren Center for Neuroscience Drug Discovery, Vanderbilt University, Nashville, TN 37232 USA; 5 Vanderbilt Kennedy Center, Vanderbilt University Medical Center, Nashville, TN 37232 USA; 6 Vanderbilt Institute of Chemical Biology, Vanderbilt University, Nashville, TN 37232 USA; 7 Department of Chemistry, Vanderbilt University, Nashville, TN 37232 USA; 8 Vanderbilt Brain Institute, Vanderbilt University, Nashville, TN 37232 USA

**Keywords:** Rett syndrome, Brainstem, Apneas, Cognition, M_1_ mAChR, Gsk3β

## Abstract

Rett syndrome (RTT) is a neurodevelopmental disorder that is characterized by developmental regression, loss of communicative ability, stereotyped hand wringing, cognitive impairment, and central apneas, among many other symptoms. RTT is caused by loss-of-function mutations in a methyl-reader known as methyl-CpG-binding protein 2 (MeCP2), a protein that links epigenetic changes on DNA to larger chromatin structure. Historically, target identification for RTT has relied heavily on *Mecp2* knockout mice; however, we recently adopted the alternative approach of performing transcriptional profiling in autopsy samples from RTT patients. Through this mechanism, we identified muscarinic acetylcholine receptors (mAChRs) as potential therapeutic targets. Here, we characterized a cohort of 40 temporal cortex samples from individuals with RTT and quantified significantly decreased levels of the *M*_*1*_, *M*_*2*_, *M*_*3*_, and *M*_*5*_ mAChRs subtypes relative to neurotypical controls. Of these four subtypes, *M*_*1*_ expression demonstrated a linear relationship with *MeCP2* expression, such that *M*_*1*_ levels were only diminished in contexts where MeCP2 was also significantly decreased. Further, we show that M_1_ potentiation with the positive allosteric modulator (PAM) VU0453595 (VU595) rescued social preference, spatial memory, and associative memory deficits, as well as decreased apneas in *Mecp2*^+/-^ mice. VU595’s efficacy on apneas in *Mecp2*^+/-^ mice was mediated by the facilitation of the transition from inspiration to expiration. Molecular analysis correlated rescue with normalized global gene expression patterns in the brainstem and hippocampus, as well as increased Gsk3β inhibition and NMDA receptor trafficking. Together, these data suggest that M_1_ PAMs could represent a new class of RTT therapeutics.

**Supplementary Information:**

The online version contains supplementary material available at 10.1007/s13311-022-01254-3.

## Introduction

Loss-of-function mutations in the *Methyl-CpG-Binding Protein 2* (*MeCP2*) gene are linked to a neurodevelopment disorder known as Rett syndrome (RTT) [1]. MeCP2 is a methyl reader protein that epigenetically regulates gene transcription either positively or negatively, depending upon its binding partners [2]. Pathogenic mutations in MeCP2 primarily either destabilize the protein, disrupt its ability to bind methylated DNA, or prevent its association with the nuclear receptor corepressor (NCoR) complex [3, 4]. RTT patients exhibit a myriad of symptoms that include developmental regression, loss of communicative ability, impaired social engagement, compulsive hand wringing, and respiratory dysfunction [5, 6].

Similar to other neurological disorders, RTT therapeutic targets are traditionally identified through expression or functional analysis in *Mecp2* knockout mice. Motivated by the high failure rate of promising preclinical compounds to translate into effective therapeutics [7], we recently adopted the alternative strategy of performing transcriptional profiling in autopsy samples from RTT patients. Through this mechanism, we sought to identify potential targets that would originate from a position of translational relevance that could be back-modeled in rodents for safety and efficacy studies [8, 9]. Using this approach, we previously identified conserved disruption of mRNA from four of the five subtypes of muscarinic acetylcholine receptors (mAChRs) in nine motor cortex and six cerebellar RTT samples compared relative to age, sex, and postmortem interval matched controls [8]. As a proof of concept, we demonstrated that a positive allosteric modulator (PAM) of the mAChR subtype 4 (M_4_) improved cognitive, social, and respiratory symptom domains in *Mecp2*^+/-^ mice.

Decreased cholinergic tone is a well-established aspect of RTT pathophysiology in patients which predates the discovery of *MeCP2* as the causative gene. In RTT autopsy samples, both decreased choline acetyltransferase (ChAT) activity and radiolabeled vesamicol binding have been reported in the putamen and in the thalamus [10, 11]. Similar results are also observed in *Mecp2*^+/-^ rats, where decreased acetylcholine levels are observed at symptomatic ages while all other neurotransmitters remain unaffected [12]. Data from RTT mouse models agree with these findings, as loss of *Mecp2* in cholinergic neurons evokes comparable anxiety, motor, social, cognitive, and cardiac phenotypes to what is observed with global *Mecp2* knockout [13–15]. Notably, these phenotypes also appear to be responsive to compounds that increase cholinergic tone such as donepezil, PNU282987, and dietary choline [13, 14, 16], as well as to viral delivery of *MeCP2* to cholinergic neurons in RTT model mice [15]. These data reinforce the hypothesis that methods of targeting cholinergic neurotransmission may have utility as RTT therapeutics and align with our data in autopsy samples that support the use of compounds that specifically potentiate mAChR signaling.

The clinical development of mAChR modulators has a robust and complex history, highlighted by seminal studies in the 1980s showing efficacy in cognitive and social symptom domains with administration of the M_1_-preferring, nonselective agonist xanomeline in patients with Alzheimer’s disease (AD) [17, 18]. While hypercholinergic adverse effects plagued mAChR discovery programs, recent advancements have shown promise in clinical trials and provided renewed hope [19]. In this study, we use a series of 40 temporal cortex samples from RTT autopsies to show that decreased *M*_*1*_ mAChR expression is both highly conserved and exhibits a linear relationship with MeCP2 expression. Further, we demonstrate that administration of the selective M_1_ PAM VU0453595 (VU595) normalizes social interaction phenotypes, rescues associative and spatial learning defects, and decreases apneas in RTT model mice while producing no overt adverse effects. We also show that treatment with VU595 normalizes global gene expression patterns in *Mecp2*^+/-^ animals and rescues pathways associated with Gsk3β inhibition and NMDA receptor (NMDAR) trafficking. Together, these data support the continued development of M_1_ mAChR positive allosteric modulators as potential RTT therapeutics.

## Methods

### Study Design

Human samples were obtained from the NIH NeuroBioBank and the University of Maryland Brain and Tissue Bank under PHS contract HHSN-271–2013-00,030. The tissues were postmortem and fully de-identified and, as such, are classified as exempt from Human Subject Research regulations. Animal work was conducted under the oversight of the Loyola University and Vanderbilt University Institutional Animal Care and Use Committees.

Mice were assigned to dosing groups at random and phenotyping or molecular quantitation was either performed by a blinded researcher or by automated software. Statistics were carried out using Prism 6.0 (GraphPad) and Excel (Microsoft). All data shown represent mean ± SEM. Statistical significance between groups was determined using two-tailed unpaired or paired student’s *t* tests and one- or two-way analysis of variance (ANOVA), with Bonferroni’s or individual student’s *t* test post hoc analysis, as specified in each figure legend. Mice sacrificed for gene expression were euthanized using CO_2_ inhalation at a flow rate of 2 L per minute, as recommended by the AVMA.

### Selection of Animal Model

The selection of mouse model (*Mecp2*^+*/tm1.1 bird*^), sex, and sample size was based on the standards established by the National Institute of Mental Health and RTT research community. Heterozygous female mice were chosen to achieve construct validity, as the overwhelming majority of RTT patients are female and *MeCP2*^*mut/*+^. A robust literature also establishes RTT-like phenotypes in this model (face validity), albeit milder than what is observed in humans, and variable in both age of onset and symptom severity, due to random x inactivation [20–22]. To account for variability, we used *N* values of > 10 mice per genotype and treatment group, compared test mice to their own baseline where possible, and performed assays at 20 weeks, when phenotypes become reproducibly observed in virtually all *Mecp2*^+/-^ mice within our colony [8, 9, 23].

### RNA-cDNA Preparation

The demographics of autopsy samples used in these studies are summarized in Table S1. Briefly, Brodmann Area (BA) 38 (temporal cortex) samples were received from *N* = 40 RTT samples with an age of 22.9 ± 2.1 years and a PMI of 20.2 ± 1.5 h. *N* = 12 controls were an age of 21.8 ± 2.4 years and a PMI of 19.3 ± 2.0 h. Approximately 1 g of the temporal cortex was impact-dissociated under dry ice and then pulverized using mortar and pestle under liquid nitrogen. Total RNA and cDNA were prepared from 200 mg of tissue using standard trizol-chloroform methodology. Brain samples from 20-week-old *Mecp2*^+/-^*(tm1.1 Bird)* mice were prepared using identical methodology.

### mRNA and Protein Analysis

Quantitative real-time (qRT)-PCR was performed on BioRad CFX96 instrumentation using Thermo Fisher Assay on Demand primer–probe kits. The assay IDs used are *CHRM1* (Hs00265195_s1), *CHRM2* (Hs06634238_s1), *CHRM3* (Hs00327458_s1), *CHRM4* (Hs00265219_s1), *CHRM5* (Hs00255278_s1), and *Chrm1* (Mm00432509_s1). Human and mouse samples were normalized to the internal control *G6PD* (Hs00166169_m1; Mm00656735_g1). qRT-PCR data was analyzed using the delta-delta Ct method. Differential RNA-sequencing was performed as previously described [8]. Differentially expressed genes were processed by Reactome.org for pathway enrichment analysis, and false discovery rates were calculated as a function of mapped genes.

Human and mouse protein from 200 mg of tissue was isolated and Western blots were run as previously described [9]. Primary antibodies were used at the following concentrations: Chrm1 (1:1000, Millipore ab5164), Gsk3α (1:500, CST4337), Gsk3α S21 (1:500, CST9316), Gsk3α/β Y216/Y279 (1:500, Fisher 44604G), Gsk3β (1:500, Abcam ab93926), Gsk3a S9 (1:500, CST9323), Nmdar2a/b (1:400, Sigma AB1548), Psd-95 (1:1000, CST3450), Gapdh (1:5000, CST5174), and β-Tubulin (1:2000, CST86298). The fluorescent secondary antibodies used were goat anti-mouse 680 (1:5000, LiCor #926–68,070), goat anti-rabbit 680 (1:5000, LiCor #926–68,071), and goat anti-rabbit 800 (1:5000, Li-Cor 926-32211). Images were acquired, and fluorescence was quantified on a Li-Cor Odyssey Infrared Imaging System.

### Compound Administration and Phenotyping

In the studies described herein, 20-week-old *Mecp2*^+/-^ and *Mecp2*^+*/*+^ females were used which represent an age where consistent phenotypes are observed in our colony. Based on the pharmacokinetics and *T*_*max*_ of the molecule [24], either vehicle (10% Tween 80) or 10 mg/kg of VU0453595 (VU595) was administered to *Mecp2*^+/-^ and *Mecp2*^+*/*+^ female mice (20w) via intraperitoneal (ip) injection 30 min before the start of the phenotypic assay and/or tissue harvest. The order phenotyping was performed was open field, three-chamber social interaction, novel object recognition, whole-body plethysmography, and contextual fear conditioning, with a minimum of 5 days washout between assays.

### Behavioral Assays

#### Open Field

Spontaneous locomotion and anxiety were measured using the open field assay. Test mice were injected with either VU595 or vehicle and placed in the activity monitoring chamber. Exploratory and locomotor behavior was then monitored using Activity software to quantify beam breaks in the X, Y, and Z planes.

#### 3-Chamber Social Preference Assay

Control and test mice were placed in a standard 3-chamber apparatus and allowed to habituate for 7 min. A novel mouse (stranger 1, 6-week-old C57B6, female) that was restrained in a wire cage was then placed in one of the end chambers, and an empty cup was placed in the opposing end chamber. The test mouse was then allowed to explore all 3 chambers for 7 min before being returned to its home cage. After 1 h, the test mouse was returned to the 3-chamber apparatus, and the ability to distinguish between stranger 1 and a novel stranger (stranger 2, 6-week-old C57B6, female) was quantified for 7 min using AnyMaze™ software.

#### Novel Object Recognition Assay

Novel object recognition (NOR) was conducted as described in [25]. Briefly, test mice were placed inside a chamber with 2 identical objects (a slide box or ball) and allowed to explore for 10 min. The animals were then returned to their home cage. After 1 h, test mice were returned to the chamber a final time for 5 min, and 1 of the 2 objects was replaced with a novel object. The test was video recorded and the seconds spent directly sniffing each object were scored by a blinded reviewer. Discrimination index was defined as (*Time*_*novel*_ – *Time*_*familiar*_) / (*Time*_*novel*_ + *Time*_*familiar*_). Direct sniffing in the novel object recognition assay was defined as an approach where the mouse’s nose either made contact or came in close proximity to the object.

### Contextual Fear Conditioning

Animals were treated with VU595 and fear-conditioned on day 1 of the task, and the percent of time spent freezing was assessed 24 h later. On the conditioning day, mice were placed into an operant chamber with a shock grid (Med Associates Inc.) in the presence of a 10% vanilla odor cue. Following a 3-min habituation period, mice were exposed to two 1-s 0.7-mA foot shocks spaced 30 s apart. After 24 h, mice were placed back into the same shock chamber with a 10% vanilla odor cue, and the percent of time spent freezing during a 3-min testing period was assessed by Med Associates software.

### Whole-Body Plethysmography

Unrestrained *Mecp2*^+/-^ and *Mecp2*^+*/*+^ mice were placed in a whole-body plethysmograph recording chamber (Buxco, 2-site system) with a continuous inflow of air (1 L/min). Following a habituation period of 30 min, a baseline recording was established for 30 min. Mice were then removed from the chamber, injected with VU595 or vehicle, and reacclimated for 30 min, and respiratory measurements were made for an additional 30 min. Analysis was performed using FinePointe Research Suite (v2.3.1.9). Apneas, defined as pauses spanning 2 × the average expiratory time of the previous 2 min, were quantified using the FinePointe apnea software patch, followed by manual spot-checking of the larger data set. Only periods of motion-free recording were analyzed. All filters were applied while the researchers were blinded to the genotype and treatment group.

## Results

### M_1_ Expression is Decreased in 40 RTT Temporal Cortex Autopsy Samples

**Fig. 1:**
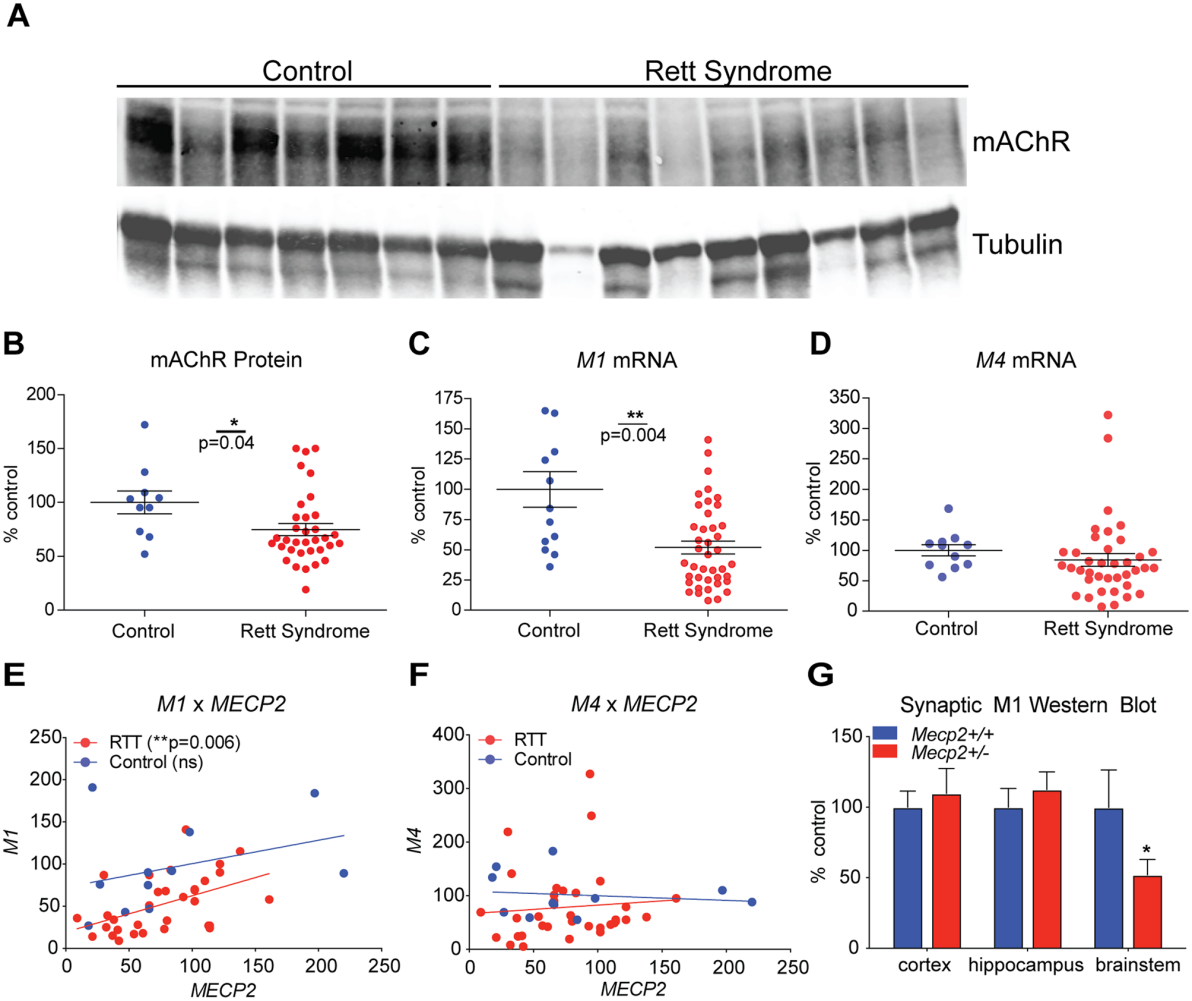
M_1_ expression is decreased in temporal cortex samples from 40 Rett syndrome (RTT) autopsy samples. **A–B** Western blot. mAChR (M_1_–M_5_) protein levels are significantly decreased in the brain samples (BA38) from RTT autopsies when compared relative to age, sex, and postmortem interval matched controls (*N* = 12). Student’s *t* test. **C–D** qRT-PCR. mRNA expression of the mAChR subtype *M*_*1*_ receptor is significantly decreased in human RTT samples, while *M*_*4*_ levels are comparable to controls in the temporal cortex. Student’s *t* test. **E–F** Linear regression analysis. A correlative analysis shows a significant linear relationship between *M*_*1*_ and *MeCP2* transcript levels, which is not observed with *M*_*4*_. Linear regression. **G** Western blot. Relative to *Mecp2*^+*/*+^ controls, synaptosome preparations from 20-week-old *Mecp2*^+/-^ mice show a significant reduction in mAChR levels in the brainstem. Two-way ANOVA with Tukey post hoc analysis. *N* = 5/treatment/genotype. *p < 0.05, **p < 0.01

Our previous work used RNA-sequencing (RNA-seq) to identify conserved disruption of mAChRs in 9 motor cortex and 6 cerebellar RTT autopsy samples [8]. While among the highest-powered transcriptional profiling studies conducted using RTT autopsy samples, the potential for variability across a larger population must be considered when determining the value of a prospective therapeutic target. To address this, we obtained 40 temporal cortex samples from patients clinically diagnosed with RTT as well as 12 age, sex, and postmortem interval matched controls (Table S1). While fully subtype-selective mAChR antibodies do not exist [26], we isolated protein and compared total (M_1_–M_5_) mAChR levels via fluorescent Western blot with an M_1_-preferring antibody. These experiments showed a significant decrease in mAChR levels in the temporal cortex of RTT patients relative to matched controls (Fig. 1A–B).

We next performed qRT-PCR to quantify mAChR subtype expression in this larger sample set. As shown in Fig. 1C and Supplemental Fig. 1, we observed a significant reduction in *M*_*1*_, *M*_*2*_, *M*_*3*_, and *M*_*5*_ expression. Remarkably, we did not see a significant change in *M*_*4*_ expression in the temporal cortex (Fig. 1D), which stands in contrast to what was observed in the motor cortex and cerebellum [8]. To determine whether the expression of any of the mAChR subtypes correlated with *MeCP2* levels, we quantified *MeCP2* mRNA expression and compared it relative to the *M*_*1*_*–M*_*5*_ mRNA levels within each patient. These experiments showed a significant linear relationship between *MeCP2* and *M*_*1*_ mRNA, such that *M*_*1*_ levels are preferentially decreased in a context in which *MeCP2* levels are also decreased (Fig. 1E–F). This relationship was not observed with any of the other mAChRs in RTT patients; however, it was observed with *M*_*2*_ and *M*_*5*_ expression in controls (Fig. S1). Although limited by the availability of human samples, using the quantified decrease in *M*_*1*_ expression, we back-calculated a value of 86.7% power for this analysis (ClinCalc), which exceeds the standard 80% used in many clinical studies.

### M_1_ Expression is Decreased in the Brainstem of Mecp2^+/-^ Mice

When coupled with the recent advancements in M_1_ positive allosteric modulator (PAM) drug discovery, the finding that M_1_ expression is decreased in the brains of RTT patients is potentially salient. To determine whether this observation also extended to mouse models of RTT, we isolated synaptosomes from the cortex, hippocampus, and brainstem of female 20-week-old *Mecp2*^+/-^ and *Mecp2*^+*/*+^ mice and performed Western blotting with an M_1_-preferring antibody. In contrast to what was observed in human samples, significantly decreased mAChR expression was only observed in the brainstem of *Mecp2*^+/-^ mice (Fig. 1G), potentially indicative of temporal, spatial, and/or species-specific factors contributing to the regulation of M_1_ expression.

### M_1_ Potentiation Rescues Social and Cognitive Deficits in Mecp2^+/-^ Mice

Given the significant decrease in M_1_ expression in clinical and preclinical sample sets, we next sought to determine whether M_1_ potentiation could improve RTT-like phenotypes in *Mecp2*^+/-^ mice. *Mecp2*^+/-^ mice and *Mecp2*^+*/*+^ littermate controls were treated with either vehicle (10% tween 80) or the M_1_-PAM VU595 and progressed through a battery of tests encompassing the major RTT symptom domains, including open field (motor and anxiety), three-chamber social interaction (social), novel object recognition (spatial memory), and contextual fear conditioning (associative memory). Mice were treated (ip) 30 min before each test, with a 5-day washout period between assays. The strategy of using a comprehensive phenotypic assessment was chosen to control for the broad expression pattern of the M_1_ receptor and the potential for VU595’s PAM activity in brain regions with normal expression to either evoke adverse effects, improve phenotypes independent of M_1_ expression, and/or influence interdependent phenotypes (i.e., anxiety and fear conditioning, motor function and sociability).

**Fig. 2:**
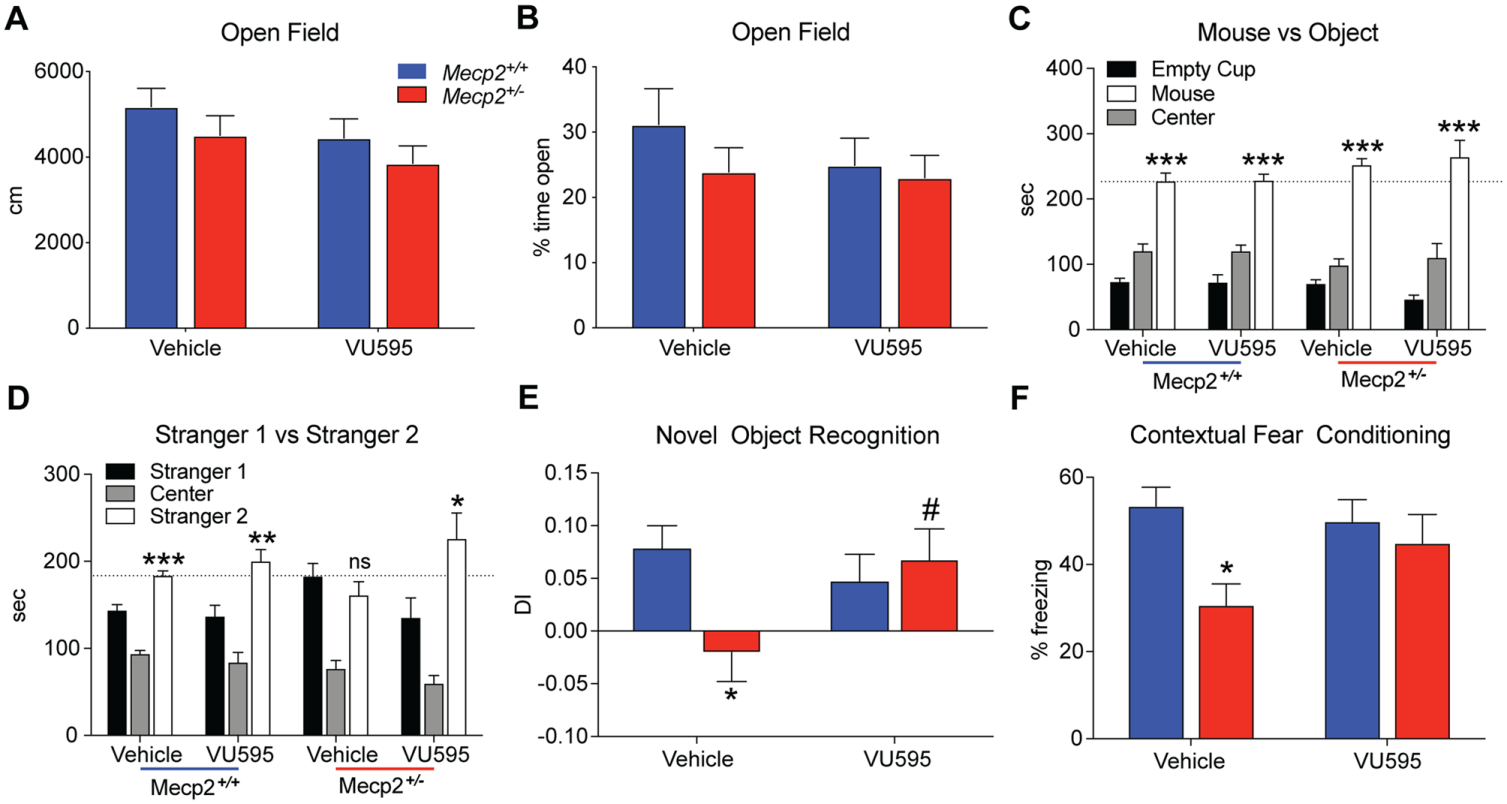
M_1_ potentiation with VU0467595 (VU595) improves social and cognitive phenotypes in the *Mecp2*^+/-^ model of Rett syndrome. *N* = 13 *Mecp2*^+*/*+^/treatment group, *N* = 11 *Mecp2*^+/-^/treatment group. **A–B** Open field. Neither genotype nor VU595 administration (10 mg/kg, ip) had an impact on spontaneous locomotion (A) and anxiety phenotypes (B) relative to vehicle *Mecp2*^+*/*+^ control mice. **C–D** Three-chamber social interaction assay. No genotype or compound effect was observed in the sociability phase of the assay (C), as all mice demonstrated a preference for the stranger 1 mouse over the empty cup. When exposed to a familiar (stranger 1) and a novel (stranger 2) mouse, *Mecp2*^+*/*+^ mice showed a preference for social novelty, independent of treatment. Conversely, vehicle-treated *Mecp2*^+/-^ mice did not distinguish between stranger 1 or stranger 2, and VU595 treatment restored preference for the novel mouse. Two-way ANOVA with Tukey post hoc analysis. **E** Novel object recognition. DI, discrimination index. Vehicle-treated *Mecp2*^+/-^ mice did not show a preference for the novel object over a familiar object, indicative of a deficit in spatial memory. VU595 administration significantly improved this phenotype in *Mecp2*^+/-^ mice. Two-way ANOVA with Tukey post hoc analysis. **F** Contextual fear conditioning. Vehicle-treated *Mecp2*^+/-^ mice presented with significantly decreased freezing when re-exposed to an aversive environment, suggesting an impairment in associative memory. Treatment with VU595 before the training phase of this assay normalized freezing in *Mecp2*^+/-^ mice to levels comparable to *Mecp2*^+*/*+^ controls. Two-way ANOVA with Tukey post hoc analysis. Ns, not significant. *p < 0.05, **p < 0.01, ***p, 0.001. #*p* < 0.05 within genotype comparison

In the open field assay, we observed no difference in spontaneous locomotion between *Mecp2*^+/-^ mice and littermate controls, regardless of treatment (Fig. 2A–B). This finding also extended to time spent in the center, where no changes were observed as a function of drug or genotype. In the three-chamber social interaction assay, there was no effect of genotype or drug on the sociability phase of the assay, with all test groups showing a preference for the stranger 1 mouse over the empty cup (Fig. 2C). Although not definitive, this finding is important in light of the sensory-perception deficits in RTT model mice [27–31], as it establishes that the capacity of test mice to detect the stranger mouse is comparable to controls. Similar to other studies [8, 9], vehicle-treated *Mecp2*^+/-^ mice failed to show a preference for the novel mouse in the second phase of this assay, while littermate controls spent significantly more time with the stranger 2 mouse, regardless of treatment (Fig. 2D). VU595 treatment significantly improved this parameter in *Mecp2*^+/-^ mice and restored social preference to levels comparable to littermate controls.

We next assessed spatial memory phenotypes using novel object recognition, where *Mecp2*^+*/*+^ controls showed significantly increased preference for the novel over the familiar object, and vehicle-treated *Mecp2*^+/-^ mice did not discriminate between the two (Fig. 2E). Following VU595 administration, there was no longer a significant difference between RTT and control test groups, indicating that spatial memory was improved. To determine whether these effects also extended to associative learning, we next progressed mice through a contextual fear assay. Following a mild foot shock, vehicle-treated *Mecp2*^+/-^ mice presented with a significant decrease in time spent freezing compared relative to vehicle-treated controls when re-exposed to the test chamber (Fig. 2F). In contrast, VU595-treated *Mecp2*^+/-^ mice were not distinguishable from controls indicative of a modest improvement in associative memory.

### VU595 Improves Respiratory Phenotypes in RTT Mice

**Fig. 3:**
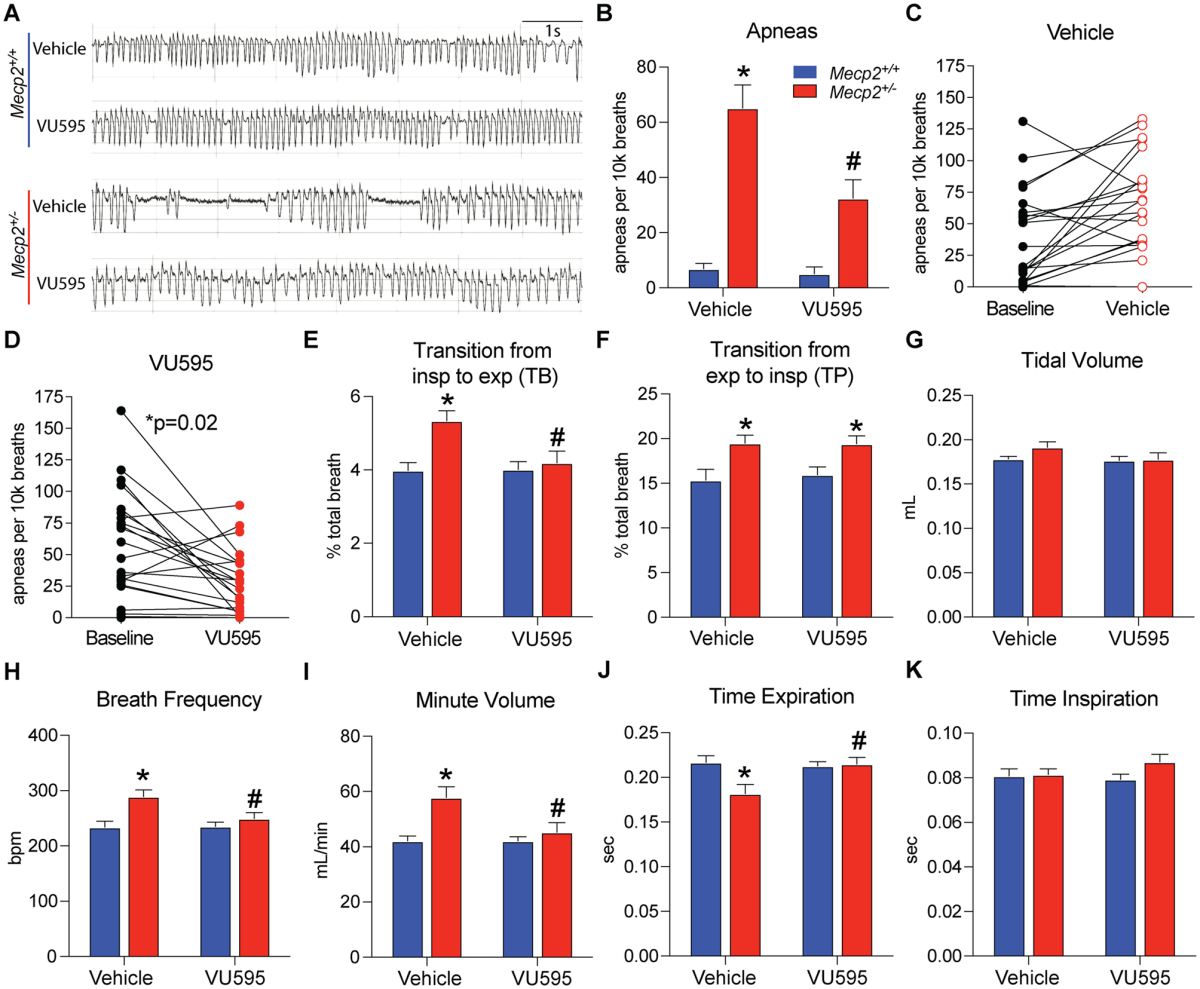
Administration of VU595 improves apneas by facilitating the transition from inspiration to expiration. Whole-body plethysmography (WBP). Unless noted, statistical comparisons were made relative to vehicle-treated *Mecp2*^+*/*+^ mice using a two-way ANOVA with Tukey post hoc analysis. *N* = 15 *Mecp2*^+*/*+^/treatment group, *N* = 22 *Mecp2*^+/-^/treatment group. **A** Representative traces of respiratory patterns. **B–D** Apneas were significantly increased in both groups of *Mecp2*^+/-^ mice; however, VU595 treatment significantly reduced the total number of apneas relative to the vehicle-treated group. A comparison relative to the baseline of each mouse shows that both groups of *Mecp2*^+/-^ mice began the experiment with comparable apnea numbers, but that only the VU595-treated group showed significant improvement. **E–F** Vehicle-treated *Mecp2*^+/-^ mice showed a significant increase in the percentage of the total breath occupied by the pause period between both inspiration and expiration. VU595’s efficacy on apneas was associated with a significant reduction in the time between inspiration and expiration, with no effect on the transition from expiration to inspiration. **G** Tidal volume was not impacted by treatment or genotype. **H–I** Breath frequency and minute volume were significantly increased in vehicle-treated *Mecp2*^+/-^ mice and were normalized by VU595 administration. **J–K** Increased breath frequency was associated with a significant decrease in expiratory time in vehicle-treated *Mecp2*^+/-^ mice, which was rescued by VU595 administration. No change in inspiratory time was observed as a function of genotype or treatment. *p < 0.05 relative to vehicle-treated *Mecp2*^+*/*+^ mice, #*p* < 0.05 relative to vehicle-treated *Mecp2*^+/-^ mice

Using the decrease in M_1_ expression in the brainstem of *Mecp2*^+/-^ mice as rationale, we next assessed VU595’s efficacy on respiratory phenotypes using whole-body plethysmography (WBP). Following 30 min of baseline recording, we administered either vehicle or VU595 and returned the mouse to the recording chamber. After trimming periods of activity from the analysis, drug effects were measured 30 min post-dose (Fig. 3A). Under this paradigm, vehicle-treated *Mecp2*^+/-^ mice exhibited significantly more apneas per 10k breaths than littermate controls, and administration of the M_1_ PAM significantly reduced the number of apneas by 49.8% (Fig. 3B). To ensure that this result was not linked to differences in random × inactivation skewing of the mutant *Mecp2* allele and/or general health differences of *Mecp2*^+/-^ test groups, we compared the number of apneas in individual mice relative to their own predrug baseline. During the baseline period, both vehicle and VU595 test groups exhibited a comparable number of apneas (Fig. 3C–D); however, following compound administration, only *Mecp2*^+/-^ mice treated with the M_1_ PAM showed a significant reduction in apnea numbers, indicative of a *bona fide* compound effect (Fig. 3D).

We next determined the location in the respiratory cycle where VU595 was impacting apneas. We examined the percent of the breath that was spent on the pause period between inspiration and expiration (TB), as well as the percent of time spent paused between expiration and inspiration (TP). In vehicle-treated *Mecp2*^+/-^ mice, both transition points occupied a significantly greater percentage of the breath relative to vehicle-treated *Mecp2*^+*/*+^ controls (Fig. 3E–F). Conversely, only TB (inspiration to expiration) was normalized by VU595, while the compound did not affect TP (expiration to inspiration). These data suggest that M_1_ potentiation decreases apneas in *Mecp2*^+/-^ mice by facilitating the transition between the inspiratory and expiratory phases of the breath cycle.

No effect of drug or genotype was recorded on tidal volume (Fig. 3G); however, we did quantify a significant increase in respiratory frequency in vehicle-treated *Mecp2*^+/-^ mice that was significantly decreased by VU595 administration (Fig. 3H). As a corollary, we also observed a significant increase in the total volume respired per minute (minute volume) in vehicle-treated *Mecp2*^+/-^ mice, which was also normalized by the M_1_ PAM (Fig. 3I). To investigate this further, we quantified the amount of time spent in the inspiration phase relative to expiration in each breath. As shown in Fig. 3J–K, the increase in breath frequency and minute volume was associated with a significantly shorter expiratory phase of the breath in vehicle-treated *Mecp2*^+/-^ mice and was normalized with VU595 treatment.

### M_1_ Potentiation Normalizes Global Gene Expression Patterns

MeCP2 is a highly abundant transcription factor in the brain that regulates gene expression locally in complex with proteins like CREB, as well as globally by linking methylated DNA with the larger chromatin architecture [2]. As a consequence, pathogenic mutations in MeCP2 result in a loosening of chromatin and a broad, but modest disruption of global gene expression patterns. M_1_ is a postsynaptic, G_q_-coupled receptor whose activation is associated with an increase in intracellular calcium and activation of calcium-dependent transcription factors [32]. To determine whether VU595 normalizes Mecp2-disrupted gene expression patterns, we administered either VU595 or vehicle to *Mecp2*^+/-^ and *Mecp2*^+*/*+^ mice, harvested the brainstem and hippocampus at 30 min post-dose, and performed differential RNA-seq analysis. The brainstem and hippocampus were chosen because they represent regions of the brain associated with apneas and cognition, where M_1_ PAM efficacy was observed. A comprehensive list of significantly disrupted genes and pathways for each brain region is provided in Supplemental Tables 2, 3, 4, 5, 6, 7, 8, and 9.

**Fig. 4:**
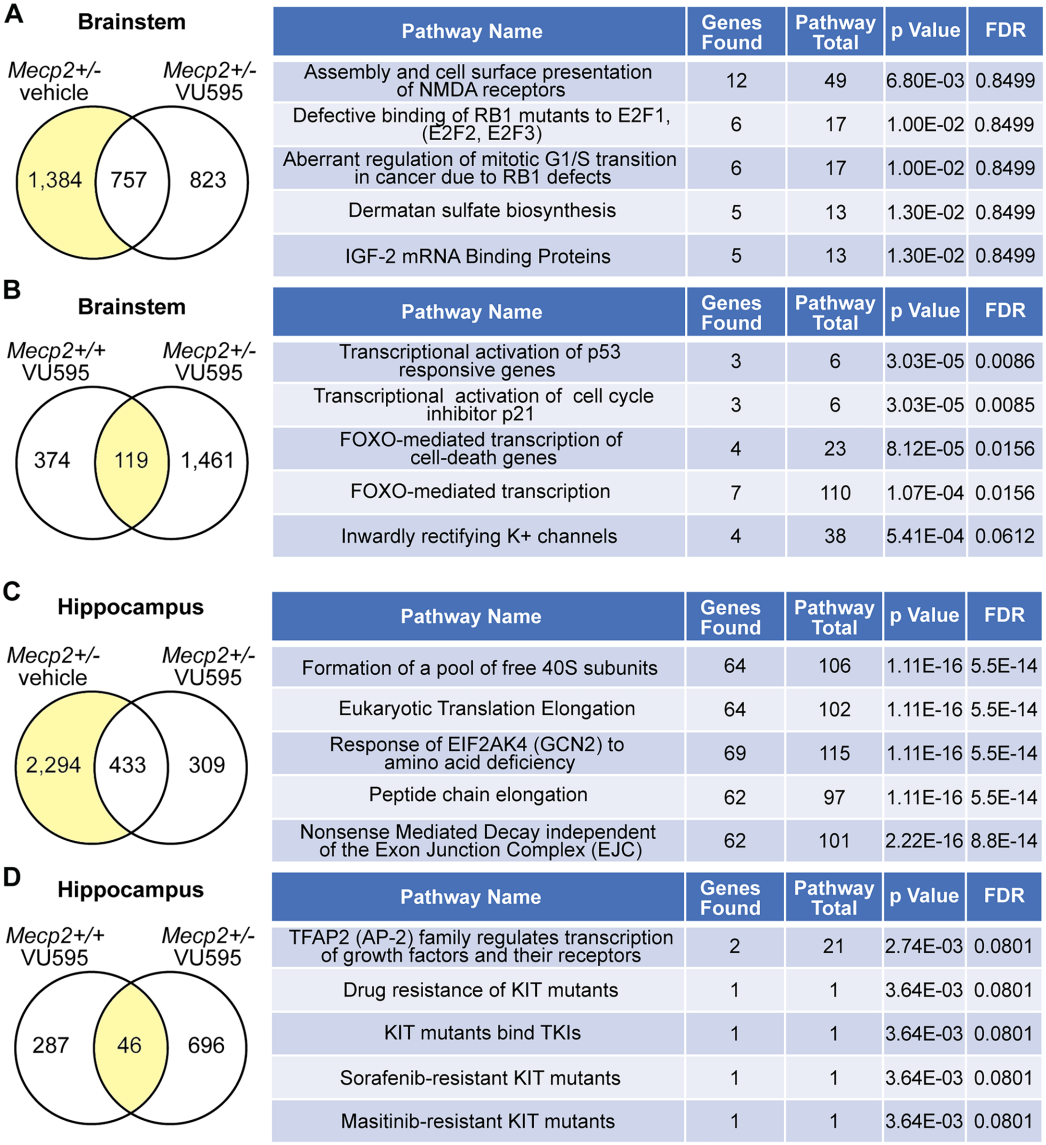
Global gene expression patterns are normalized by M_1_ potentiation. Differential RNA-sequencing relative to vehicle-treated *Mecp2*^+*/*+^ control mice followed by reactome pathway enrichment analysis. **A** Brainstem. A total of 2,141 genes were significantly disrupted in vehicle-treated *Mecp2*^+/-^ mice. Following VU595 treatment, the expression of 1,384 (64.6%) was normalized, while 757 remained disrupted. Right: pathway analysis of rescued genes. **B** Brainstem. Following VU595 administration, the expression of 119 genes was significantly altered in both *Mecp2*^+*/*+^ and *Mecp2*^+/-^ mice. Right: pathway analysis of conserved genes. **C** Hippocampus. A total of 2,727 genes were significantly disrupted in vehicle-treated *Mecp2*^+/-^ mice. Following VU595 treatment, the expression of 2,294 (84.1%) was normalized, while 433 remained disrupted. Right: pathway analysis of rescued genes. **D** Hippocampus. Following VU595 administration, the expression of 46 genes was significantly altered in both *Mecp2*^+*/*+^ and *Mecp2*^+/-^ mice. Right: pathway analysis of conserved genes

In the brainstem, vehicle-treated *Mecp2*^+/-^ mice had 2,141 differentially expressed genes (DEGs) relative to vehicle-treated *Mecp2*^+*/*+^ samples, and 1,384 (64.7%) of those genes were no longer disrupted in mice treated with VU595 (Fig. 4A). Reactome.org (v76) analysis of the normalized genes showed enrichment in pathways associated with assembly and cell surface presentation of NMDARs, among others. We next examined genes that were significantly changed by VU595 treatment in both *Mecp2*^+*/*+^ and *Mecp2*^+/-^ mice, which identified 119 genes that were affected in both genotypes (Fig. 4B). Pathway analysis of these genes points to an enrichment in FOXO/P21/P53 signaling, which has previously been associated with pathogenic mutations in MeCP2 that disrupt NCoR/HDAC3 binding [33].

In total, 2,727 DEGs were quantified in the hippocampus of vehicle-treated *Mecp2*^+/-^ mice, and 2,294 (84.1%) of those genes were no longer disrupted in *Mecp2*^+/-^ mice treated with VU595 (Fig. 4C). Reactome.org analysis of the normalized genes showed enrichment in pathways associated with the regulation of nonsense-mediated decay and formation of the 40S ribosomal subunit, among many other pathways. The expression of 46 genes exhibited conserved disruption by VU595 in both *Mecp2*^+*/*+^ and *Mecp2*^+/-^ mice, which demonstrated no significant pathway enrichment (Fig. 4D).

**Fig. 5:**
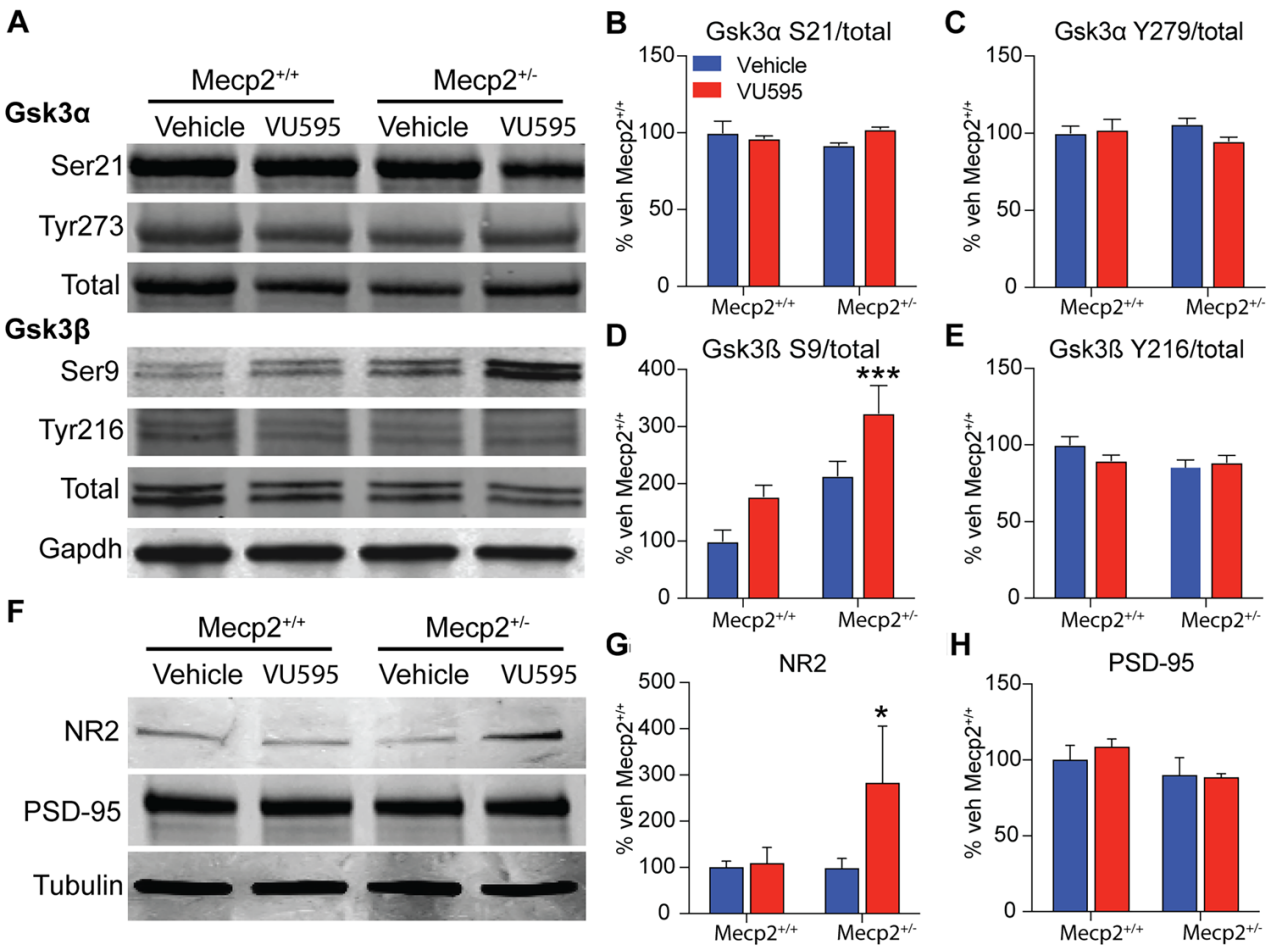
M_1_ potentiation increases both Gsk3β inhibition and synaptic NMDAR levels in the brainstem of *Mecp2*^+/-^ mice. **A** Representative Western blots from the brainstem of *Mecp2*^+*/*+^ and *Mecp2*^+/-^ mice treated with vehicle or VU595. **B–C** Neither the inhibition (S21) nor the activation (Y279) sites on Gsk3α were significantly impacted by treatment with VU595 as measured by the ratio of phosphorylated (P) to total (T) protein. **D** Administration of VU595 significantly increased the P/T ratio at the S9 inhibitory site of Gsk3β, specifically in the brainstem of *Mecp2*^+/-^ mice, while having no impact in *Mecp2*^+*/*+^ mice. Two-way ANOVA with Tukey post hoc analysis. **E** The activation site (Y216) of Gsk3β was not affected in the brainstem by VU595 treatment, regardless of genotype. **F** Representative synaptosome Western blots from the brainstem of *Mecp2*^+*/*+^ and *Mecp2*^+/-^ mice treated with vehicle or VU595. **G** VU595 significantly increased the presence of NR2a/b containing NMDARs at the synapse of *Mecp2*^+/-^ mice while having no impact on *Mecp2*^+*/*+^ control mice. Two-way ANOVA with Tukey post hoc analysis. **H** PSD-95 control Western blots confirm that synaptosomes assessed in these experiments contained postsynaptic structures in equivalent amounts across treatments and genotypes. *p < 0.05, ***p < 0.001

In addition to calcium-dependent gene regulation, M_1_ activation impacts the activation of calcium-independent kinases [34], many of which are integral to maintaining proper synaptic transmission. In light of the brainstem RNA-seq and whole-body plethysmography data, one kinase of interest was Gsk3β, which is known to regulate NMDAR trafficking [35] and whose inhibition has been shown to reduce respiratory phenotypes in RTT model mice [36]. To determine whether M_1_ potentiation was impacting Gsk3β-signaling, we treated *Mecp2*^+*/*+^ and *Mecp2*^+/-^ mice with VU595, isolated protein from the brainstem, and performed Western blotting to profile the inhibitory (S9, S21) and activation (Y216/Y279) phosphorylation sites on Gsk3β and its homolog, Gsk3α. Contrary to previous reports [36], we observed no difference in Gsk3β or Gsk3α phosphorylation in *Mecp2*^+/-^ mice relative to vehicle-treated controls at either the inhibitory or activation sites (Fig. 5A–C). Further, neither the inhibitory nor activation sites of Gsk3α were impacted by VU595 administration. Conversely, VU595 treatment significantly increased phosphorylation of the Gsk3β S9 inhibitory site in VU595-treated *Mecp2*^+/-^ mice relative to vehicle-treated *Mecp2*^+*/*+^ controls, while the activation site was unaffected (Fig. 5D–E). These data suggest that M_1_ potentiation promotes inhibition of Gsk3β signaling in the brainstem of RTT model mice, which may contribute to the rescue of respiratory phenotypes independent of a primary deficit in Gsk3β-signaling.

To determine whether changes in Gsk3β S9 phosphorylation correlated with a change in NMDAR presence at synapses, we isolated PSD95 containing synaptosomes from the brainstem of mice treated with either vehicle or VU595. We then performed Western blotting and probed using a nonselective NR2 subunit antibody. As shown in Fig. 5F–H, there was no difference between vehicle-treated *Mecp2*^+*/*+^ and *Mecp2*^+/-^ mice; however, VU595 treatment significantly increased the presence of NMDARs at the synapse of *Mecp2*^+/-^ mice relative to controls. While further work is required to establish the functional consequences of increased NMDARs specifically at respiratory nuclei, these data suggest that increased M_1_/Gsk3β/NMDAR signaling in the brainstem correlates with the decrease in apneas in RTT model mice induced by the M_1_ PAM VU595.

## Discussion

Recent clinical development campaigns have shown remarkable progress toward potentially viable Rett syndrome therapeutics [37]. To improve the rigor of our research, we have embraced a reverse-translational approach to target identification. These efforts identified a conserved disruption in mAChRs in RTT autopsy samples [8]. Here, we confirm those findings using a cohort of 40 temporal cortex samples from RTT patients and demonstrate that four of the five mAChRs are significantly decreased in expression. Further, we show that positive allosteric modulation of the M_1_ receptor normalizes social preference, associative and spatial memory deficits, and respiratory phenotypes in *Mecp2*^+/-^ mice, a model with face and construct validity for the clinical disorder [23, 37].

Among our most interesting findings is that *M*_*1*_ expression correlated directly with *MeCP2* expression in autopsy samples, such that *M*_*1*_ levels are only low when *MeCP2* levels are also low. Given that not all pathogenic mutations impact *MeCP2* gene or protein expression (i.e., missense mutations), these data suggest that decreased *M*_*1*_ expression may be enriched in patients with truncating and/or destabilizing mutations [38]. Our sample set precludes testing this hypothesis, as it consists of patients who were clinically diagnosed with RTT, but never genotyped for *MeCP2* mutations. As such, further studies with larger sample sizes are required to substantiate whether a relationship between *MeCP2* mutation and *M*_*1*_ expression exists.

The M_1_ receptor has a complicated history as a potential therapeutic target for neurological disorders that is highlighted by the clinical efficacy of the M_1_/M_4_-preferring agonist xanomeline in Alzheimer’s disease [17, 18]. The overwhelming majority of subsequent drug discovery efforts were derailed preclinically by the presence of either gastrointestinal or convulsive adverse effects [39]. In recent years, both of these adverse effects have been largely mitigated through selective targeting of the M_1_ receptor with positive allosteric modulators that are void of agonist activity [40–42] and have low cooperativity profiles [43, 44] or by co-dosing xanomeline with a peripherally restricted pan-muscarinic receptor antagonist [19]; however, it bears mentioning in this context since RTT patients are prone to gastrointestinal complications and seizures [45, 46]. We did not observe either phenotype in our studies with *Mecp2*^+/-^ mice, although it remains possible that they might emerge with chronic dosing.

The compound used in our studies, VU595, has previously been shown to exhibit efficacy in correcting abnormal social and spatial memory phenotypes in a chronic phencyclidine (PCP) model of schizophrenia [24], and our data in *Mecp2*^+/-^ mice parallels these findings. Specifically, acute administration of VU595 restored social memory and/or preference in RTT mice without impacting sociability in the three-chamber social interaction assay. Impaired spatial and associated memory phenotypes were also rescued by M_1_ potentiation in the novel object recognition and contextual fear assays, respectively. These results align with M_1_’s expression pattern, where enrichment is observed at the prefrontal cortical and hippocampal circuits that largely govern these behaviors.

There is a well-established role for mAChRs in the control of breathing [47]; however, this is generally associated with M_2_ and M_3_ receptor expression in the autonomic nervous system and not with M_1_ receptors. Given M_1_’s limited-expression pattern in the brainstem, it was an unexpected result that VU595 administration normalized respiratory phenotypes in *Mecp2*^+/-^ mice. While increased breath rate has previously been reported in *Mecp2*^+/-^ mice [48], here we link this finding in vehicle-treated mice with a decrease in expiratory time. Equally as important as the impact of VU595 on breath rate is its capacity to decrease apneas, which our data link to facilitation of the transition from inspiration to expiration. This finding agrees with in situ physiology data showing that increased post-inspiratory duration is associated with hyperexcitation of expiratory inputs originating in the Kölliker-Fuse nucleus in *Mecp2*^*−/y*^ mice [49]. In agreement with these data, glutamate microinjection at these inputs can evoke apneas in ex vivo preparations from the same model [48, 50]. Accordingly, our data may suggest that M_1_ potentiation may decrease apneas by tempering hyperexcitation in the Kölliker-Fuse nucleus, although this will need to be tested experimentally.

Our RNA-sequencing experiments established that one of the top pathways that was rescued in the brainstem of VU595-treated *Mecp2*^+/-^ animals involves the assembly and trafficking of NMDARs, and follow-up studies implicate Gsk3β inhibition in mediating the observed efficacy. NMDA-signaling has a well-developed history in RTT research, where both deletion of the NR2A subunit [28] and administration of the NMDAR antagonist ketamine rescue multiple aspects of RTT pathophysiology in mice [51–53]. Previous studies have also shown that Gsk3β inhibition rescues multiple symptom domains, including respiration [36], and is known to regulate NMDAR activity and trafficking, in part, via activation of phosphatidylinositol 4 kinase type II α (PI4KIIα) [35]. NMDAR activation has been shown to dephosphorylate the S9 inhibitory site of Gsk3β [54], thereby providing a mechanistic point of convergence for ketamine, VU595 (M_1_ PAM), and SB216763 (Gsk3β inhibitor) to act on apneas in the brainstem of RTT model mice. One important aspect of our data quantifying Gsk3β inhibition is that it was not significantly affected in the brainstem of vehicle-treated *Mecp2*^+/-^ mice, and trended towards an increased baseline, as has previously been reported in other brain regions (36). This suggests that insufficient Gsk3β inhibition is likely not the driver of respiratory phenotypes. When analyzed in concert with the SB216763 data, one potential unifying explanation is that Gsk3β inhibition is a compensatory mechanism that cannot reach the required levels to maintain proper respiratory function in the absence of Mecp2 due to decreased M_1_-expression and signaling. If true, it would suggest that VU595 and SB216763 function by enhancing an existing neuroprotective mechanism to regulate synaptic NMDAR levels; however, further work is required to determine if this is a *bona fide* mechanism of action.

 In summary, we describe the use of a large cohort of temporal cortex autopsy samples from RTT patients to establish the rationale for the M_1_ receptor as a potential therapeutic target. Drug discovery campaigns for positive modulators of mAChRs have made significant advancements in recent years, such that compounds with favorable drug-like properties now exist. Using the M_1_ PAM tool compound VU595, we observed efficacy in social, cognitive, and respiratory symptom domains in *Mecp2*^+/-^ mice. Phenotypic improvement was correlated with both normalization of gene expression and increased inhibition of Gsk3β. Together, these data advocate for continued development and optimization of M_1_ PAMs for RTT, as well as for related autism-associated disorders with overlapping pathologies.

## Supplementary Information

Below is the link to the electronic supplementary material.

**Supplemental Table 1:** Demographics of Rett syndrome autopsy samples used for mAChR expression profiling. Supplementary file1 (XLSX 12 KB)

Supplementary file2 (XLSX 387 KB): Disrupted Genes in Branstem *Mecp2*^+/-^ vehicle vs *Mecp2*^+/-^ VU595

Supplementary file3 (XLSX 24.5 KB): Pathways disrupted in the brainstem of *Mecp2*^+/-^ mice treated with vehicle relative to *Mecp2*^+/-^ mice treated with VU595

Supplementary file4 (XLSX 256 KB): Disrupted Genes in Brainstem *Mecp2*^+/+^ VU595 vs *Mecp2*^+/-^ VU595

Supplementary file5 (XLSX 25 KB): Pathways disrupted in the brainstem of both *Mecp2*^+/-^ mice treated with VU595 and *Mecp2*^+/+^ mice treated with VU595

Supplementary file6 (XLSX 355 KB): Disrupted Genes in Hippocampus *Mecp2*^+/-^ vehicle vs *Mecp2*^+/-^ VU595

Supplementary file7 (XLSX 47 KB): Pathways disrupted in the hippocampus of *Mecp2*^+/-^ mice treated with vehicle relative to *Mecp2*^+/-^ mice treated with VU595

Supplementary file8 (XLSX 157 KB): Disrupted Genes in Hippocampus *Mecp2*^+/+^ VU595 vs *Mecp2*^+/-^ VU595

Supplementary file9 (XLSX 19 KB): Pathways disrupted in the hippocampus of both *Mecp2*^+/-^ mice treated with VU595 and *Mecp2*^+/+^ mice treated with VU595

**Supplemental Figure 1. *M***_***2,3,5***_ **expression is decreased in temporal cortex samples from Rett syndrome (RTT)autopsies. A-C)** qRT-PCR analysis. *M*_*2*_, *M*_*3*_, and *M*_*5*_expression is significantly decreased in temporal cortex samples from RTT patient autopsies when compared relative to age, sex, and post-mortem intervalmatched controls. Students t-test. *p<0.05, ***p<0.001. **D-F)** Linear regression. Unlike *M*_*1*_ (Figure 1), *M*_*2*_, *M*_*3*_, and *M*_*5*_expression did not correlate with *MeCP2 *expression in RTT patient samples; however, a comparable linear relationshipwas observed with *M*_*2*_ and *M*_*5*_ expression in matched controls. Note that due to limited quantities of autopsy samples, *M*_*2*_, *M*_*3*_, and *M*_*5 *_expression were assessed using N=24 RTT samples and N=11 matched controls. Linear regression. **p<0.01,***p<0.001. Supplementary file10 (TIF 881 KB)

Supplementary file11 (PDF 435 KB)

Supplementary file12 (PDF 508 KB)

Supplementary file13 (PDF 1443 KB)

Supplementary file14 (PDF 517 KB)

Supplementary file15 (PDF 508 KB)

Supplementary file16 (PDF 508 KB)

Supplementary file17 (PDF 509 KB)

Supplementary file18 (PDF 518 KB)

Supplementary file19 (PDF 436 KB)

Supplementary file20 (PDF 517 KB)
