# Supplementary figures and images for: Clinical and Preclinical Evidence for M1 Muscarinic Acetylcholine Receptor Potentiation as a Therapeutic Approach for Rett Syndrome
Source: Neurotherapeutics. 2022 Jun 7;19(4):1340–52. doi: 10.1007/s13311-022-01254-3 (PMC9587166; doi:10.1007/s13311-022-01254-3)

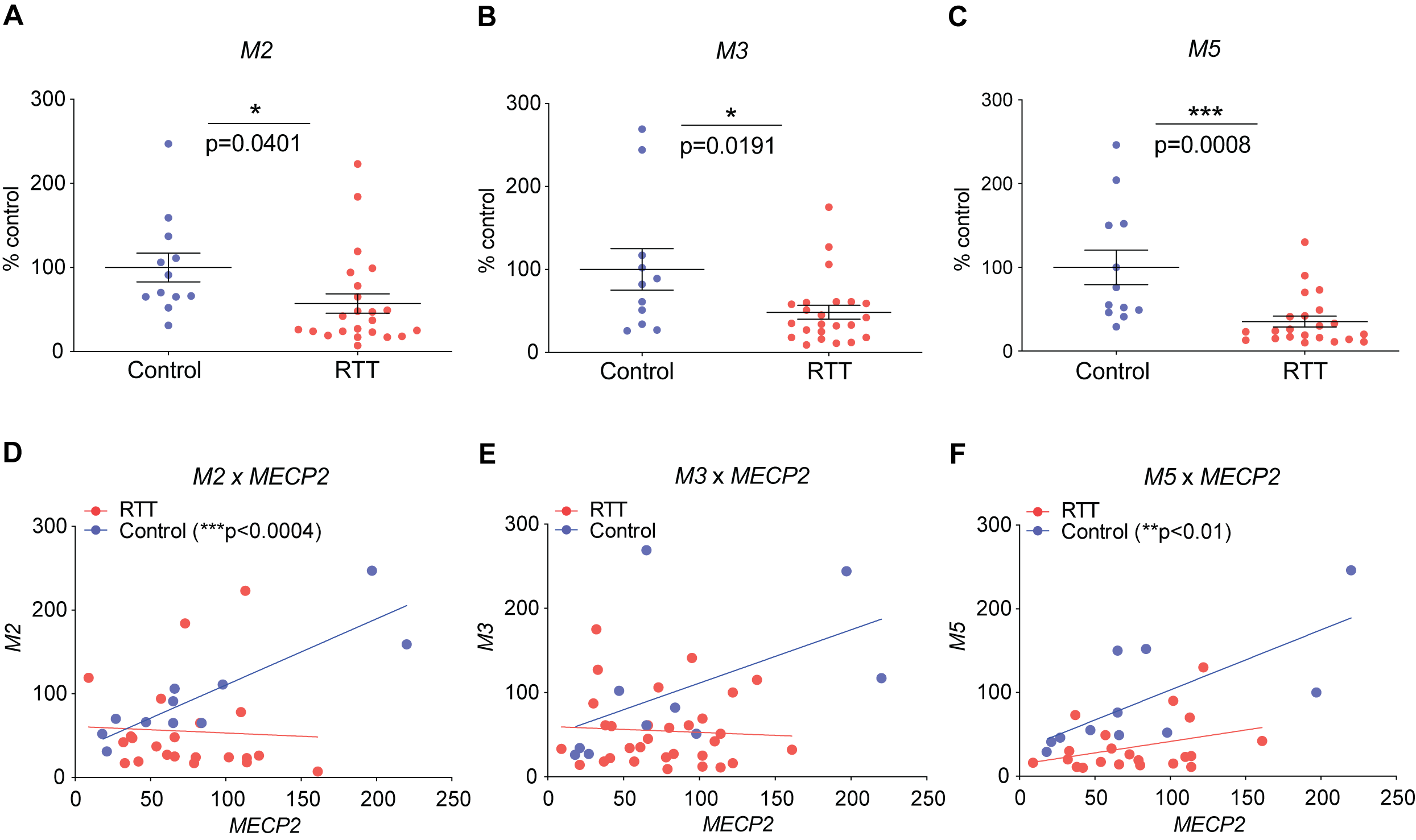

Supplement: Supplementary file 10 — Supplemental Figure 1. M2,3,5 expression is decreased in temporal cortex samples from Rett syndrome (RTT)autopsies. A-C) qRT-PCR analysis. M2, M3, and M5expression is significantly decreased in temporal cortex samples from RTT patient autopsies when compared relative to age, sex, and post-mortem intervalmatched controls. Students t-test. *p<0.05, ***p<0.001. D-F) Linear regression. Unlike M1 (Figure 1), M2, M3, and M5expression did not correlate with MeCP2 expression in RTT patient samples; however, a comparable linear relationshipwas observed with M2 and M5 expression in matched controls. Note that due to limited quantities of autopsy samples, M2, M3, and M5 expression were assessed using N=24 RTT samples and N=11 matched controls. Linear regression. **p<0.01,***p<0.001. Supplementary file10 (TIF 881 KB) [file 13311_2022_1254_MOESM10_ESM.tif]
